# Pheromones and Barcoding Delimit Boundaries between Cryptic Species in the Primitive Moth Genus *Eriocrania* (Lepidoptera: Eriocraniidae)

**DOI:** 10.1007/s10886-019-01076-2

**Published:** 2019-05-31

**Authors:** Jean-Marc Lassance, Glenn P. Svensson, Mikhail V. Kozlov, Wittko Francke, Christer Löfstedt

**Affiliations:** 1 Department of Biology, Lund University, Sölvegatan 37, SE-22362 Lund, Sweden; 2 Present Address: Department of Organismic and Evolutionary Biology, Harvard University, 16 Divinity Avenue, Cambridge, MA 02138 USA; 3 Section of Ecology, Department of Biology, University of Turku, FI-20014 Turku, Finland; 4 Institute of Organic Chemistry, University of Hamburg, Martin-Luther-King Platz 6, 20146 Hamburg, Germany

**Keywords:** Cryptic species, Speciation, Pheromone signalling, Barcoding, COI, GADPH, Wingless

## Abstract

Animal classification is primarily based on morphological characters, even though these may not be the first to diverge during speciation. In many cases, closely related taxa are actually difficult to distinguish based on morphological characters alone, especially when there is no substantial niche separation. As a consequence, the diversity of certain groups is likely to be underestimated. Lepidoptera –moths and butterflies– represent the largest group of herbivorous insects. The extensive diversification in the group is generally assumed to have its origin in the spectacular radiation of flowering plants and the resulting abundance of ecological niches. However, speciation can also occur without strong ecological divergence. For example, reproductive isolation can evolve as the result of divergence in mate preference and the associated pheromone communication system. We combined pheromone trapping and genetic analysis to elucidate the evolutionary relationships within a complex of primitive moth species (Lepidoptera: Eriocraniidae). Mitochondrial and nuclear DNA markers provided evidence that *Eriocrania semipurpurella*, as currently defined by morphological characters, includes three cryptic species in Northern and Western Europe. Male moths of these cryptic species, as well as of the closely related *E. sangii*, exhibited relative specificity in terms of their attraction to specific ratios of two major pheromone components, (2*S*,6*Z*)-nonen-2-ol and (2*R*,6*Z*)-nonen-2-ol. Our data suggest strong assortative mating in these species in the absence of apparent niche separation, indicating that *Eriocrania* moths may represent an example of non-ecological speciation. Finally, our study argues in favour of combining pheromone investigations and DNA barcoding as powerful tools for identifying and delimitating species boundaries.

## Introduction

At times of global biodiversity loss, with populations of many species suffering from reductions or disappearance of their habitats (Barnosky et al. 2011), cataloguing the diversity of life remains of fundamental importance. Current estimates of the number of leaves on the Tree of Life are affected by the way in which traditional taxonomists have defined boundaries between groups of organisms to classify them into species. Although morphological changes are not always and necessarily associated with the process of speciation (Svensson 2012), the vast majority of taxonomic resources available to date rely mainly on specimens’ morphological traits to delineate species (Bickford et al. 2007). As a consequence, many biological species may be hidden under a single nominal species description. These so-called cryptic species have few to no diagnosable and unambiguous differentiating morphological characters. This is a consequence of limited time since they diverged from a common ancestor or morphological stasis imposed by stabilizing selection maintaining a particular morphology (Bickford et al. 2007). They may, however, have diverged in their ecology and/or reproductive biology, and might thus be recognized only once their existence has been revealed most often by means of DNA sequence analysis. DNA barcoding has provided the tools to expand our recognition of species beyond the classical boundaries set by morphological information (Hebert et al. 2003).

The Lepidoptera –moths and butterflies– is one of the most taxonomically speciose insect orders and certainly one group that has received much attention by taxonomists (Dinca et al. 2011; Mutanen et al. 2010; van Nieukerken et al. 2011). Speciation is the major evolutionary process generating species diversity and studying how animals evolve reproductive isolation helps us to understand both the process of speciation and the nature of species differences (Butlin et al. 2012). In the Lepidoptera, rapid radiation paralleled the spectacular diversification in flowering plants in the Early Cretaceous (Misof et al. 2014), providing a vast number of new niches for this largely herbivorous insect group. However, although there are examples of radiation in the absence of niche separation in Lepidoptera (Imada et al. 2011), the role of non-ecological speciation might be underestimated in this animal group as well as in other taxa (Svensson 2012). One aspect of lepidopteran biology that may have favoured diversification in the absence of ecological niche differentiation might reside in their mate-finding system, which is largely based on chemical communication signals, also known as pheromones. In moths especially, subtle chemical changes in sex pheromone signalling may be the initial trigger for population divergence and can lead to reproductive isolation and contribute to the speciation process (Allison and Cardé 2016; Cardé and Haynes 2004; Löfstedt 1993; Smadja and Butlin 2009). At the same time, chemical traits such as pheromones often do not represent prominent characters in our classification of the natural world. Given that cryptic species are often differentiated by nonvisual mating signals, and thus do not require visually detectable morphological divergence (Bickford et al. 2007), species that have diverged by means of changes in their communication system may be mistakenly considered a single species.

**Fig. 1:**
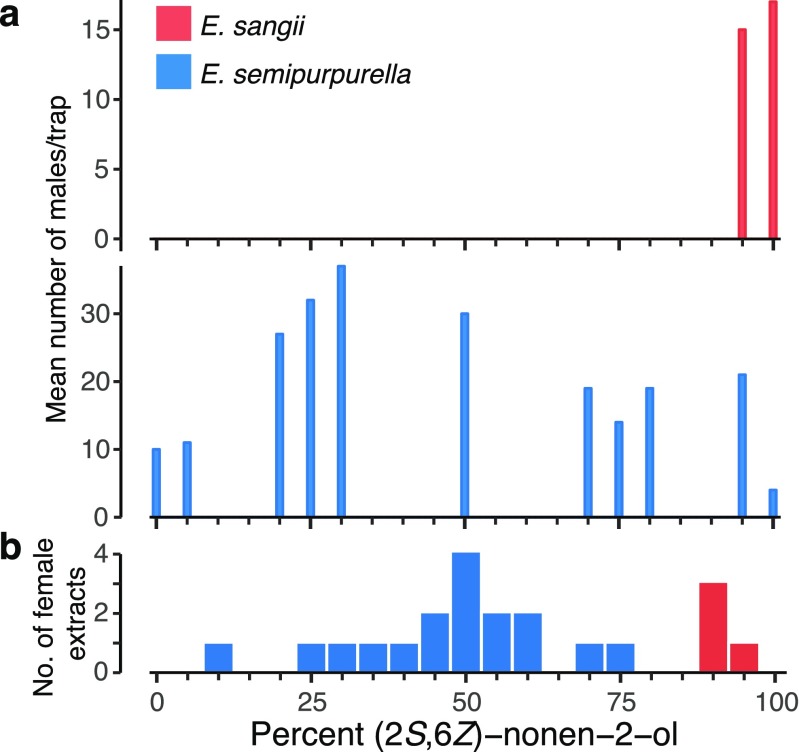
Natural variation in male response and female pheromone composition in the *Eriocrania semipurpurella*-*sangii* complex in Turku (Finland). **a** Mean number of male moths trapped with baits containing eleven different enantiomeric ratios of (2*R*,6*Z*)-nonen-2-ol and (2*S*,6*Z*)-nonen-2-ol, as observed in field tests conducted in 1994 (redrawn after Kozlov et al. 1996). **b** Enantiomeric composition of pheromone extracts from individual females as determined by enantioselective gas chromatography. Species assignment was based on morphological character

In this study, we integrate molecular (mitochondrial and nuclear DNA markers) and ethological data (attraction to synthetic pheromone baits) to study the *Eriocrania semipurpurella-sangii* species complex (Lepidoptera: Eriocraniidae). These archaic moths are univoltine leafminers using birch (*Betula* spp.) as exclusive host (Koricheva and Haukioja 1995). *Eriocrania* females produce a pheromone consisting of a blend of short-chain alcohols and ketones to attract potential mates over a distance (Kozlov et al. 1996; Larsson et al. 2002; Zhu et al. 1995). In particular, the two major pheromone components, (2*S*,6*Z*)-nonen-2-ol and (2*R*,6*Z*)-nonen-2-ol, have been shown to be sufficient to trigger attraction of males to baited traps under field conditions. Variation in the attractiveness of different enantiomeric ratios has been reported previously (Kozlov et al. 1996). Males attracted to lures containing 95% to 100% of the (*S*)*-*enantiomer are typically identified as *E. sangii* (Wood), whereas individuals associated with other ratios are identified as *E. semipurpurella* (Stephens) based on current identification keys (Heath 1976; Kozlov 1997; Sutter 2000). However, there seems to be substantial geographical variation among the response profiles of individuals identified as *E. semipurpurella*. For example, Kozlov and colleagues (Kozlov et al. 1996) reported that the distribution of trap catches in Sweden, Finland and Russia differed, suggesting the existence of populations with different pheromone composition. In particular, the distribution of trap catches among baits with different enantiomeric composition in Finland appeared to not have a unimodal distribution (Fig. 1a), suggesting the coexistence of different populations*.* These data, along with anecdotal reports of temporal variation in flight phenology as well as variation in male genitalia morphology (Karsholt O, Kozlov MV, Kristensen NP, personal communication) has led to the hypothesis that the species morphologically defined as *E. semipurpurella* may actually consist of a complex of sibling species. To address this question, we analysed the pheromone composition of individual females and sampled males at different geographic locations using pheromone trapping. We then genotyped males at mitochondrial and nuclear molecular markers and asked whether moths of the *E. semipurpurella-sangii* complex which are attracted to different blends represent distinct genetic clusters across different populations in Northern and Western Europe.

## Methods and Materials

### Pheromone Synthesis

Pheromone synthesis followed the approach described earlier (Kozlov et al. 1996). As determined by enantioselective gas chromatography (25 m fused silica capillary, coated with a 1:4 mixture of 6-O-methyl-2,3-di-O-pentyl-γ-cyclodextrin and OV-1701, run at 80 °C), (2*R*,6*Z*)-nonen-2-ol was obtained in 97% overall purity and 99.7% enantiomeric excess (ee), whereas (2*S*,6*Z*)-nonen-2-ol showed an overall purity of 96% and 99.5% ee.

### Re-Analysis of Male Response Profile

Kozlov et al. (1996) reported differences in male response profiles among geographic populations of *E. semi-purpurella* and an apparent bimodality in the response profile observed in Turku, Finland (Fig. 1a). The latter statement, however, had not been formally tested. We thus decided to evaluate indications of deviation from unimodality of the male trapping data using two different measures of bimodality, namely the bimodality coefficient (BC) (SAS Institute 1990), which is interpreted as bimodal for values >0.555, and Hartigans' dip statistic (HDS) (Hartigan and Hartigan 1985). These two methods rely on different properties to evaluate the data distribution, and convergence in the two measures can be used to detect the presence of bimodality (Freeman and Dale 2013). Hartigans’ dip statistic and the bimodality coefficient were implemented using the R packages *diptest* version 0.75–7 (Maechler 2016) and *mousetrap* version 3.1.2 (Kieslich et al. 2019), respectively.

### Chemical Analyses

To determine the extent of variation in female pheromone composition, moths were collected as adults using net sweeping in young birch forests near Turku (Finland) in May 1997. Individuals were sexed and all females were saved for pheromone extraction. The form of the terminal segment of the long maxillary palp of *E. semipurpurella* and *E. sangii*, exhibit a pointed apex or a clear bifurcation, respectively. This morphological character was used to discriminate between the two taxa. We dissected the fifth abdominal segment from adult females and extracted them individually for at least 30 min in a pointed glass tube containing 20 μl redistilled heptane. These extracts were subsequently used for chemical analyses using the aforementioned enantioselective gas chromatography conditions.

### Sample Collection

Male *Eriocrania* moths from six geographical locations were collected in the course of pheromone field-trapping experiments conducted in birch forests during the spring season (April–June) (Table 1). Synthetic pheromone blends were prepared in hexane. For each lure, a 100-μl aliquot containing 100 μg of active ingredient(s) was loaded on a red rubber septum (Arthur H. Thomas Co. # 1780-J07 in 1997–2002; Wheaton # 224100–020 in 2010). Blend ratios are listed in Table 1. Using delta traps, the pheromone trapping experiments were performed as described by Kozlov et al. (1996). Individual moths were recovered from the sticky insert of pheromone-baited traps and placed in microcentrifuge plastic tubes containing ethanol (95%).

**Table 1:** Information about *Eriocrania* samples used in the present study

Site	Country	Coordinates	Pheromone baits^1,2^	Number of individual screened		
				*COI*	*Wingless*	*GADPH*
Kirovsk 1997^3^	Russia	67.64 N, 33.94 E	a	6	3	4
			g	12	5	5
Turku 1997	Finland	60.46 N, 22.25 E	a	15	5	3
			b	14	6	6
			c	19	1	1
			d	20	1	1
			e	20	4	3
			f	20	9	9
			g	19	2	2
Uppsala 2010	Sweden	59.86 N, 17.64 E	a	2	2	2
			b	12	3	2
			c	7	1	1
			d	7	1	1
			e	10	5	5
			f	12	9	8
			g	7	2	2
Lund 1996	Sweden	55.71 N, 13.19 E	a	1	1	1
			b	13	1	1
			c	14	1	1
			d	13	1	1
			e	10	1	1
			f	10	3	2
			g	12	2	1
London 2002	United Kingdom	51.52 N, 0.13 W	a	2	1	1
			d	7	3	3
			g	3	2	2
Arlon 2010	Belgium	49.68 N, 5.78 E	a	0	0	0
			b	11	3	3
			d	14	2	2
			f	3	3	3
			g	7	3	3

^1^Pheromone baits consisted of blends of (2*R*,6*Z*)-nonen-2-ol and (2*S*,6*Z*)-nonen-2-ol (a: 100/0; b: 95/5; c: 75/25; d: 50/50; e: 25/75; f: 5/95; g: 0/100). ^2^ Blends that are not listed were not used in field experiments. ^3^ Kozlov et al. (1996) reported that intermediate blends are not attractive at this location

### DNA Extraction, Amplification and Sequencing

The mitochondrial marker *cytochrome oxidase subunit I* (*COI*) was sequenced from 322 specimens, and the nuclear markers *Wingless* and *Glyceraldehyde 3-phosphate dehydrogenase* (*GADPH*) from 86 and 80 specimens, respectively. In addition, sequences from two individuals of *E. cicatricella* (Zetterstedt) were obtained and used as outgroup.

Genomic DNA was extracted from ethanol-preserved eriocraniid moths using the Qiagen DNeasy tissue kit (Qiagen) following the manufacturer’s instructions. DNA amplification was performed using the polymerase chain reaction (PCR) using the following primers: for *COI* LepF1 (5’-ATTCAACCAATCATAAAGATATTGG-3′) (Hebert et al. 2004) and Nancy (5’-CCCGGTAAAATTAAAATATAAACTTC-3′) (Simon et al. 1994) or LCO (5’-GGTCAACAAATCATAAAGATATTGG-3′) and HCO (5’-TAAACTTCAGGGTGACCAAAAAATCA-3′) (Folmer et al. 1994); for *Wingless* LepWg1 (5’-GARTGYAARTGYCAYGGYATGTCTGG-3′) and LepWg2 (5’-ACTICGCARCACCARTGGAATGTRCA-3′) (Brower and DeSalle 1998); for *GADPH* Frigga (5’-AARGCTGGRGCTGAATATGT-3′) and Burre (5’-GWTTGAATGTACTTGATRAGRTC-3′) (Wahlberg and Wheat 2008). PCRs were carried out in a final volume of 15 μl containing 1 μl of extracted DNA, 0.1 μl of AmpliTaq Gold polymerase (5 U/μl; Applied Biosystems), 1.5 μl of Gold PCR buffer (Applied Biosystems), 3 or 1.5 μl of 25 mM MgCl_2_ (*COI* or *Wingless* and *GADPH*; Applied Biosystems), 0.3 μl of each primer (10 μM), 0.3 μl of dNTP mix (10 mM; Fermentas), and 8.5 or 10 μl of sterile water. PCR profiles were as follow: for *COI*: 5 min at 95 °C, 40 cycles of 30 s at 95 °C, 40 s at 46 °C, 2 min at 72 °C, and 10 min at 72 °C; for *Wingless* and *GADPH*: 5 min at 95 °C, 40 cycles of 60 s at 95 °C, 60 s at 58 °C, 2 min at 72 °C, and 10 min at 72 °C.

PCR products were treated with a mixture containing Exonuclease I (EXO; Fermentas) and Shrimp Alkaline Phosphatase (SAP; Fermentas) in order to remove unincorporated dNTPs and primers. EXO/SAP treated products were sequenced in both directions using the corresponding gene-specific primers with the BigDye terminator kit version 1.1 (Applied Biosystems) followed by analysis on a ABI PRISM 3130XL genetic analyzer (Applied Biosystems). Forward and reverse sequences were manipulated with Bioedit version 7.0 (Hall 1999), and the sequence information combined into contigs. Any heterozygous position was coded following the IUPAC ambiguity code.

### Phylogenetic Analyses

The phylogenetic relationships among moths were inferred using maximum parsimony and Bayesian methods. For *COI*, we condensed the dataset to a non-redundant collection of haplotypes with DNASP 5 (Librado and Rozas 2009). For *Wingless* and *GADPH*, we used the extended datasets.

Maximum parsimony analyses were performed in PAUP 4.0a123 (Swofford 2002) using full heuristic search and default parameters (starting trees obtained using stepwise addition; addition sequence simple; tree-bisection-reconnection (TBR) as branch-swapping algorithm; multiple trees in effect). Bootstrap values were obtained by performing 1000 replicates.

Prior to Bayesian analyses, we determined the most appropriate model of nucleotide substitution to use with our molecular datasets employing the program TOPALI v2.5 (Milne et al. 2009). Following model selection based on the Akaike Information Criterion (AIC), phylogenetic reconstruction was inferred using MrBayes v3.2 (Ronquist and Huelsenbeck, 2003) and carried under the GTR+ Γ model for *COI* and *Wingless* and SYM + Γ for *GADPH*. The Bayesian analysis was performed for 1 million generations (first 25% discarded as burnin) and every 100th generation was sampled.

We constructed a maximum parsimony network with the mitochondrial data using TCS version 1.21 (Clement et al. 2000). This statistical parsimony method with a 95% connection limit can be used for identifying species boundaries when applied to non-recombining loci such as *COI* (Hart and Sunday 2007).

To derive information on species distributions, we compiled a dataset comprised of our DNA barcoding data together with data from GenBank and BOLD databases. Specifically, we recovered samples identified as *Eriocrania* spp. and for which the metadata contained information about the sampling location. In total, we recovered 310 sequences corresponding to same region of the *COI* gene as our data. The combined 375 nucleotide sequences were aligned using MAFFT (Katoh and Standley 2013) and the resulting alignment used to infer a phylogenetic tree in MrBayes using the parameters specified above for *COI*. The resulting tree was used to determine group membership to the Genbank and BOLD sequences. A total of 181 sequences could be assigned to one of the clades including the non-redundant haplotypes identified in this study. We used the R package *ggmap* for data visualization (Kahle and Wickham 2013).

### Estimation of Divergence Times

Divergence times were estimated using the *COI* gene in MrBayes using a strict clock model. The dataset was partitioned so that the three codon positions were treated separately to accommodate heterogeneity in the evolutionary process across sites and estimate parameters independently. We used the conventional 2.3% per million years mutation rate for the arthropod *COI* gene (Brower 1994), which corresponds to a substitution rate of 0.0115 substitutions per site per million year. Two independent MCMC analyses were run for 1 million generations with Markov chains sampled every 100 generations.

### Summary Statistics

We used DNASP 5 (Librado and Rozas 2009) to perform polymorphism analyses and calculate the following summary statistics: number of haplotypes (*h*), haplotype diversity (*H*_*d*_), π and θ nucleotide diversity. These descriptive parameters were estimated for the entire dataset and for data subsets corresponding to the genetic clusters identified by our gene genealogies reconstruction. We calculated Tajima’s *D* statistic (Tajima 1989) to detect possible departures from a neutral equilibrium model for each locus and clade separately.

## Results

Kozlov et al. (1996) reported differences in male response profiles among geographic populations of *E. semipurpurella* and an apparent bimodality in the distribution of trap catches observed in Turku, Finland (Fig. 1a). The re-analysis of the response profiles by two different methods allowed us to reject the hypothesis about the unimodality of the distribution (Bimodality Coefficient = 0.588; Hartigans’ dip statistic, *p* value <0.001). We then compared the male response profile to the distribution of female pheromone phenotypes to get a sense of the extent of variation in female pheromone composition in the same population. Our analyses revealed, in spite of the limited sample size, that females in the population indeed produce a wide range of pheromone blend ratios (Fig. 1b).

**Fig. 2:**
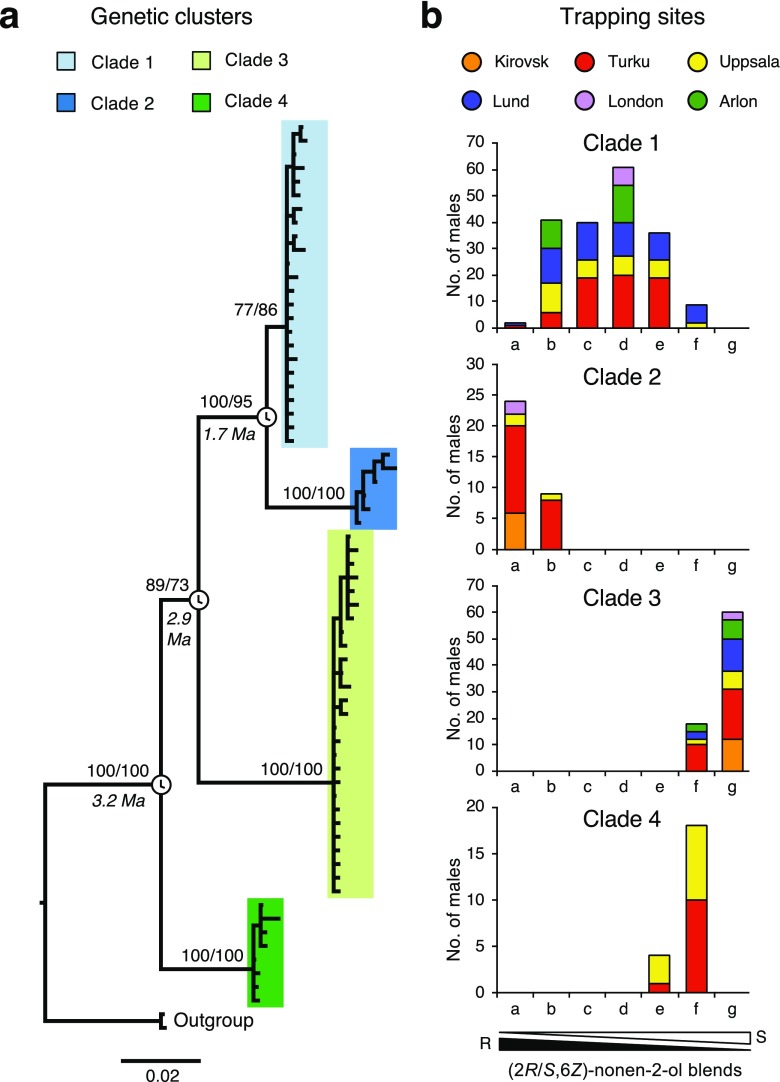
Genetic differentiation among male *Eriocrania* moths in relation to pheromone attraction. **a** mtDNA phylogenetic tree showing the relationships among 65 non-redundant *COI* haplotypes derived from *Eriocrania semipurpurella*-*sangii* populations in the Western Palearctic region. Four well-supported clades are identified. Probability values shown above branches were estimated using Bayesian analysis and maximum parsimony bootstrap support, respectively. Mean estimated ages for the speciation events are indicated at the corresponding nodes. The scale bar represents the number of substitutions per site. **b** Distributions of the number of genotyped individual male moths belonging to each of the four identified genetic clusters classified after the pheromone blends the males were attracted to. The pheromone baits consisted of seven serial blends of (2*R*/2*S*,6*Z*)-6-nonen-2-ol (a: 100/0; b: 95/5; c: 75/25; d: 50/50; e: 25/75; f: 5/95; g: 0/100). Colour represents a particular trapping location. Note the different scales of the y-axes

These results together with the documented geographic variation in male response profiles prompted us to examine further the possible genetic differentiation existing between animals attracted to different pheromone blends. For *COI,* we generated a total of 322 sequences that were 658 bp in length and revealed 103 polymorphic sites (78 parsimony-informative characters) and 65 unique haplotypes. The *COI* region amplified exhibited a high level of diversity (Table 2). Our results indicate that multiple genetic groups exist within the *E. semipurpurella-sangii* complex. Based on the inspection of the *COI* Bayesian and MP trees and the associated bootstrap values, we identified four major clades with strong statistical support (Fig. 2a). These clades did not distinguish haplotypes based on geographic origin but rather reflect differences in the pheromone baits to which the males were attracted (Fig. 2b). Clade 1 consisted of samples from Belgium, England, Finland and Sweden and included individuals attracted to intermediate blends of (2*R*,6*Z*)-nonen-2-ol and (2*S,6Z*)-nonen-2-ol. Clade 2 was sister to clade 1 and is attributable to individuals attracted to blends containing 100% to 95% of (2*R*,6*Z*)-nonen-2-ol. These specimens were collected in the Kola Peninsula (Russia), Finland, mid-Sweden and England. Clade 3 consisted of samples from all sampled locations and comprised exclusively male moths attracted to baits composed at 100% to 95% of (2*S*,6*Z*)-nonen-2-ol. The last cluster (clade 4) is composed of individuals trapped only in Finland and mid-Sweden within traps baited with blends containing 25/75 and 5/95 of (2*R*,6*Z*)-nonen-2-ol and (2*S,6Z*)-nonen-2-ol, respectively.

**Table 2:** Genetic diversity and summary statistics for each clade

Locus	Clade	*n*	*S*	*h*	*H*_*d*_	*π*	*θ*	Tajima’s *D*^2^
*COI*	1	189	26	24	0.539	0.00120	0.00679	−2.30317**
	2	33	8	6	0.426	0.00308	0.00337	−0.26213
	3	78	57	28	0.874	0.00518	0.01851	−2.37696**
	4	22	10	8	0.827	0.00240	0.00417	−1.44592
	all	322	103	65	0.825	0.03637	0.02872	
*Wingless* ^1^	1	32	6	7	0.505	0.00145	0.00285	−1.19086
	2	14	3	3	0.320	0.00120	0.00173	−0.73650
	3	25	6	9	0.713	0.00390	0.00301	0.74950
	4	15	2	3	0.467	0.00112	0.00113	−0.02430
	all	86	47	22	0.875	0.03296	0.01924	
*GADPH* ^1^	1	31	5	6	0.645	0.00119	0.00157	−0.55587
	2	13	3	4	0.674	0.00126	0.00116	0.21604
	3	23	5	6	0.636	0.00115	0.00168	−0.76779
	4	13	1	2	0.148	0.00022	0.00039	−0.71385
	all	80	62	18	0.888	0.03224	0.01721	

*n*: number of sequences; *S*: total number of polymorphic sites; *h*: number of haplotypes; *H*_*d*_: haplotype diversity; *π*: pi nucleotide diversity; *θ*: theta nucleotide diversity

^1^We determined the phase of nuclear genotypes using the program Phase implemented in DNASP 5

^2^Significance of Tajima’s *D*: ** *P <* 0.01

**Fig. 3:**
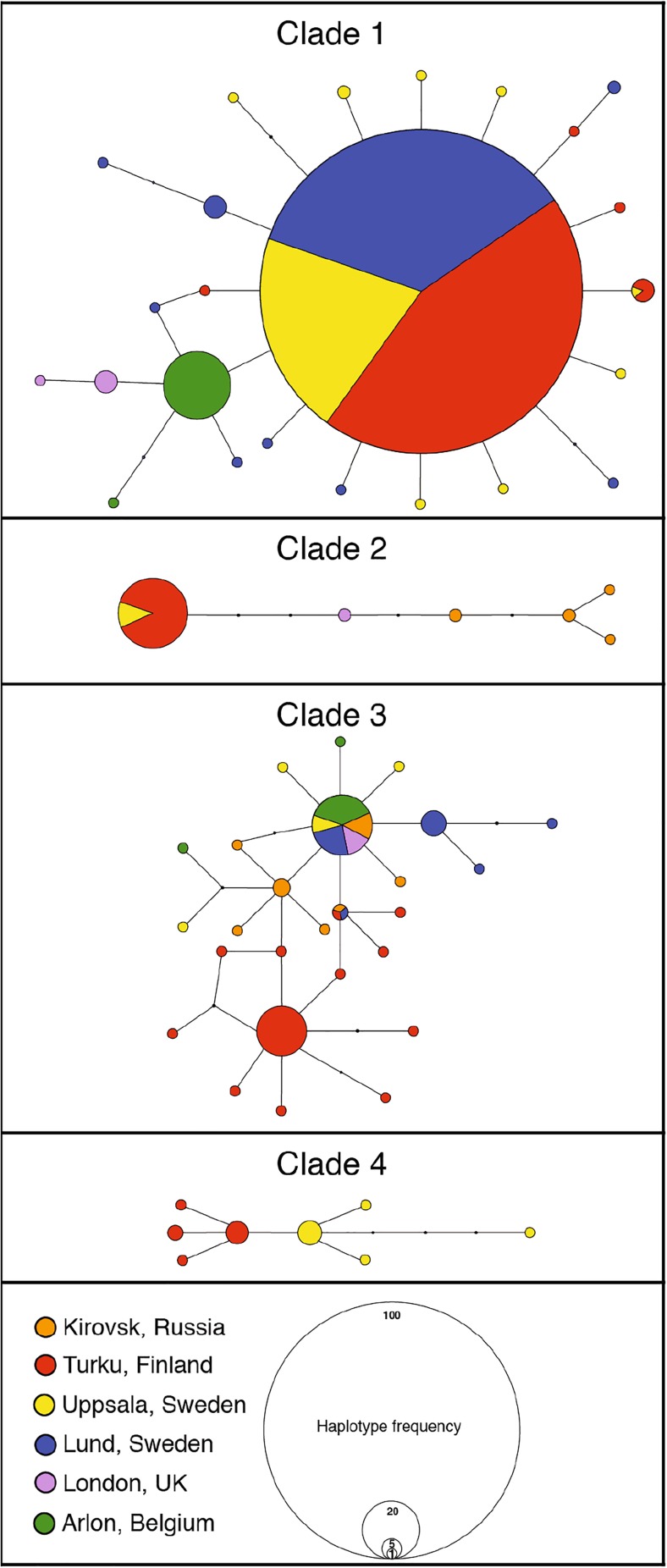
Statistical parsimony haplotype subnetworks resulting from the analysis of *COI*. The area of each circle is proportional to the frequency of a particular haplotype and nodes represent unsampled or extinct haplotypes. Colours correspond to localities

Based on morphological characters, we identified clade 3 as *E. sangii*, whereas clades 1, 2 and 4 were all identified as *E. semipurpurella*. In concordance with the phylogenetic analysis, the statistical parsimony analysis of *COI* produced four independent subnetworks (Fig. 3), confirming the monophyly of each clade. Several haplotypes are shared among several geographic locations.

**Fig. 4:**
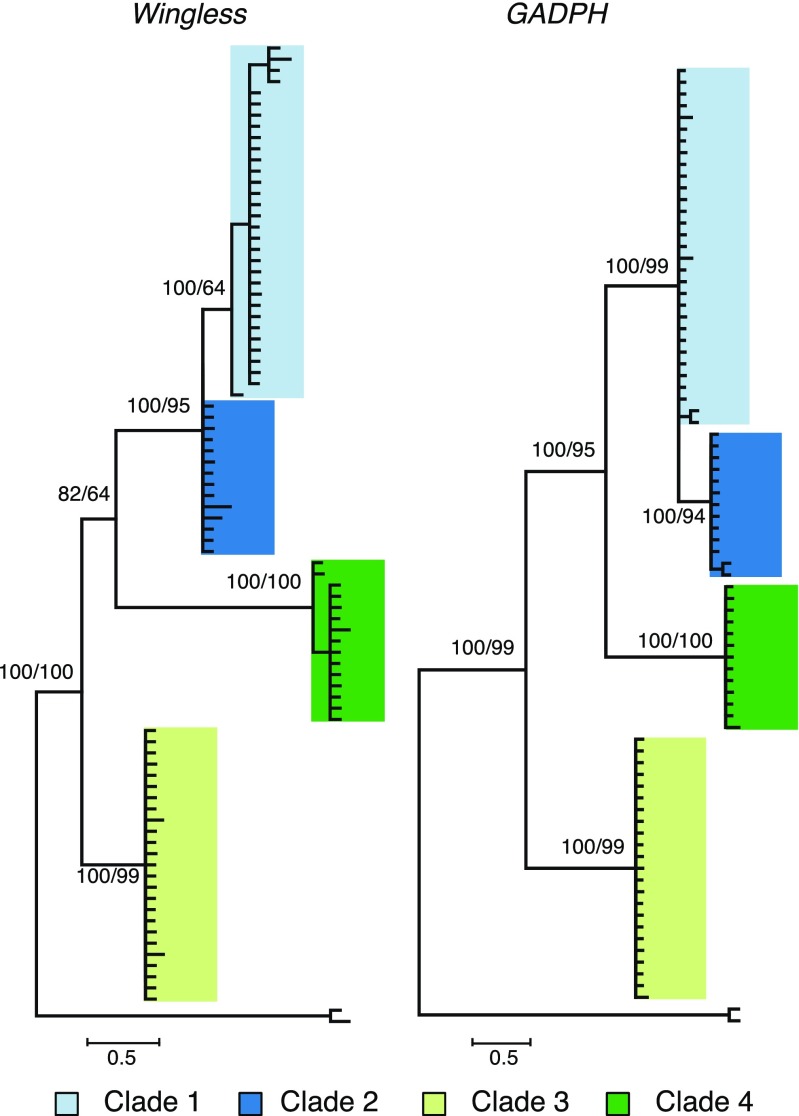
Genealogical trees based on the nuclear markers *Wingless* and *GADPH*. Bayesian posterior probabilities and maximum parsimony bootstrap support are indicated above branches

**Fig. 5:**
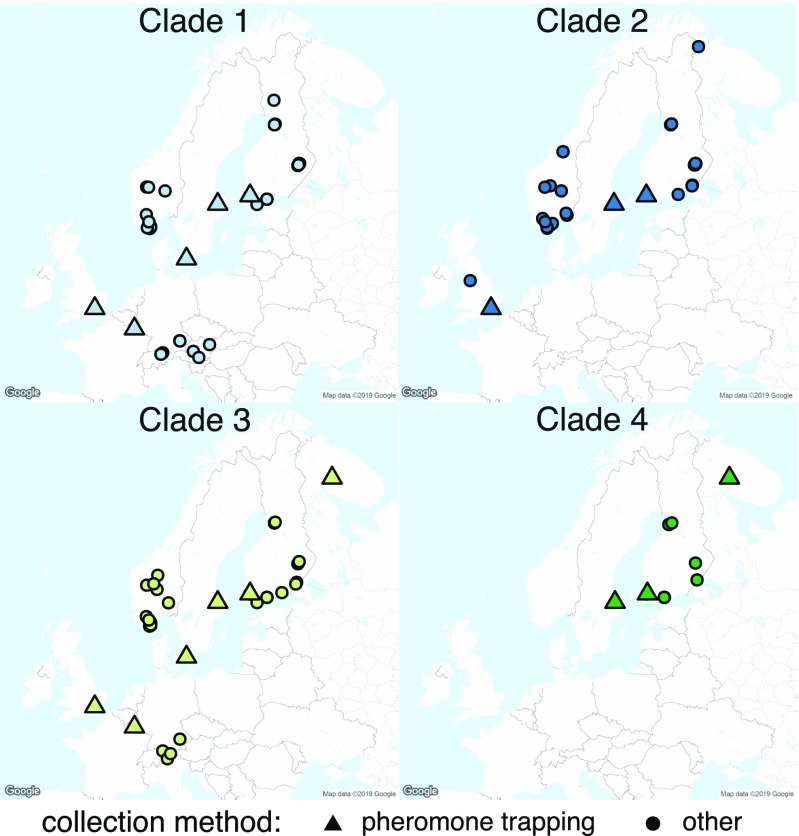
Locations of the Western and Northern part of Europe where moths from each clade of the *Eriocrania semipurpurella-sangii* complex were collected as part of this study or other barcoding studies (pheromone trapping and other, respectively)

The deep splits at the mtDNA level are corroborated by divergence at the nuclear loci monitored here. For amplification of the nuclear loci (*Wingless* and *GADPH*), we selected a subset of samples from each clade representative of all geographic origins. The Bayesian and maximum parsimony gene genealogies estimated for *Wingless* and *GADPH* appear largely congruent with the *COI* tree, with the exception of an inversion between clade 3 and clade 4 in the gene tree topologies (Fig. 4). Although most groups exist in sympatry in several locations (Fig. 5), we did not find any case of introgression between genetic clusters at the sampled loci.

Pairwise genetic differentiation among clades was extensive. The level of sequence divergence among the four genetic clades ranged from 3.92 to 7.70% for *COI,* for *Wingless* ranged from 0.49 to 5.58% and ranged from 0.63 to 5.24% for *GADPH* (Table 3). The mean divergence between clades was always much higher than the divergence intraclade (Table 3). Nucleotide diversity varied between the four clades (Table 2) but was not correlated with sample size (Pearson’s correlation coefficient, r = −0.44, *n* = 4, *P =* 0.56). The neutral equilibrium model was generally upheld. Negative values of Tajima’s *D* summary statistics observed with clade 1 and 3 (Table 2) indicate an excess of low frequency haplotypes. Times estimated using a strict clock model and a divergence rate of 2.3% per million years indicate that the species complex diversified during the Pliocene-early Pleistocene (Fig. 2). The initial split of clade 4 from the other clades happened during the Pliocene (95% highest posterior density interval: 1.72–5.14 Mya), and later, clade 3 diverged at estimated times extending from 1.52 to 4.57 Mya. Clade 1 and 2 diverged in the Pleistocene (HPD: 0.77–2.78 Mya).

**Table 3:** Mean uncorrected sequence divergence within and between clades

*COI*		1	2	3	4
	1	*0.0012*			
	2	0.0392	*0.0031*		
	3	0.0653	0.0749	*0.0036*	
	4	0.0643	0.0737	0.0770	*0.0024*
*Wingless*		1	2	3	4
	1	*0.0002*			
	2	0.0049	*0.0003*		
	3	0.0372	0.0326	*<0.0001*	
	4	0.0513	0.0510	0.0558	*0.0003*
*GADPH*		1	2	3	4
	1	*0.0002*			
	2	0.0063	*0.0004*		
	3	0.0494	0.0521	*<0.0001*	
	4	0.0391	0.0524	0.0422	*0.0002*

## Discussion

The present study provides answers to long-standing taxonomic questions concerning the *E. semipurpurella-sangii* complex. Differences in the response profiles of *E. semipurpurella* males from different localities to blends of (2*R*,6*Z*)-nonen-2-ol and (2*S,6Z*)-nonen-2-ol were reported previously (Kozlov et al. 1996). We demonstrate that the morphologically-defined species *E. semipurpurella* consists of three genetically distinct, reproductively isolated, species. Our data suggest that pheromonal differences are likely to be an important factor contributing to reproductive isolation through the usage of distinct communication channels.

Although limited due to the inherent difficulties to collect females in the field, our chemical analyzes suggest that the wide range of blend ratios that can attract *E. semipurpurella* is mirrored to a large extent by variation in the composition of the female pheromone (Fig. 1a and b). In pheromone communication systems, overlap between emitter and recipient is usually observed but rarely perfect (Allison and Cardé 2016; Löfstedt 1993). Indeed, males may be attracted to blends of pheromone components well outside what females typically produce. Further sampling would be required to obtain an exhaustive representation of the variation in female pheromone production. Collecting males in pheromone-baited traps, on the other hand, is a more amenable task. Therefore, we were prompted to examine further the significance of the bimodality in the male response profile seen in Finland. Taken together, our data revealed that individuals attracted to different blends of (2*R*,6*Z*)-nonen-2-ol and (2*S,6Z*)-nonen-2-ol are actually genetically differentiated.

The divergence between the clades identified in this study is well above a threshold of 3% divergence for *COI* suggested for accurate delineation of lepidopteran species (Hebert et al. 2003). Because analysing the matriline alone (mitochondrial DNA) may result in over-estimation of the genetic clustering (Dasmahapatra et al. 2010), it is important to observe concordance between mitochondrial and nuclear markers, as reflected by our data. In the present case, phylogenetic analyses across three loci as well as statistical parsimony analysis at the mitochondrial gene *COI* provide strong support for genetic clusters in the form of four taxonomic groups of a similar taxonomic rank within the *E. semipurpurella-sangii* complex in Northern and Western Europe. Using the well-accepted mutation rate of 2.3% per Ma for the mitochondrial gene *COI* in Lepidoptera (Brower 1994; Sperling and Nazari 2007; Wahlberg 2006), the age estimates we obtained suggest that the complex is a young group, probably diverging during the Late Miocene and Pleistocene. Our data suggest that the different pheromone types display partially overlapping geographical distributions, with some types being found at all sampled locations and others more restricted in their distribution. This is corroborated by samples collected as part of other barcoding surveys (Fig. 5), suggesting that the absence of a species at a given trapping site may be seen as evidence that it is absent in the corresponding region. More sampling would be necessary to establish the definite range of these species.

We relied on pheromone-baited traps to sample the individuals analysed in this study. This strategy allows for collecting medium to large numbers of individuals of the targeted taxa with relatively high specificity (few to no other species are found in the traps). Also, it can provide additional information about the behavioural response profile of the taxa analysed. Generally speaking, female moth pheromones act as a long-distance attractant, and males make a choice of potential partners based on the information conveyed by the pheromone signal (Löfstedt 1993). Using pheromone-baited traps is a way of assessing the extent of male preference for particular signals in a competitive context and the use of different pheromone blends as potential premating reproductive barrier. Our results show that males from each of the clades delineated by molecular markers exhibit a specific response profile, providing information about the likely composition of their pheromones. Assessing the pheromone composition of females combined with genotyping would, of course, provide final confirmation of among-clade differences in pheromone communication. Although our pheromone trapping experiments did not provide information on time of peak flight activity, we can reasonably hypothesize that pheromone differences play a significant role in limiting interactions between these sympatric species. Hybridisation between *Eriocrania* species is likely limited as a consequence of differences in their communication channel and mate-finding behaviour. Indeed, we did not detect any hybrid individuals among our samples, although this conclusion awaits further confirmation using a genome-wide approach and examining more loci.

The sampling strategy employed here was not compatible with the preservation of voucher specimens that would allow parallel examination of morphological characters. However, a number of *Eriocrania* males were collected using insect net as they approached pheromone baits, and these specimens could be used to identify morphological differences between the clades, together with voucher specimens for which barcoding information is available. The taxonomic descriptions of the *Eriocrania* species representing the unnamed clades will be published elsewhere (Kozlov MV, Mutanen M, Karsholt O, personal communication). Barcoding is a powerful tool to reveal some of the hidden diversity existing in nature, even in well-studied groups. Yet, barcoding alone does not provide biological explanation to understand what contributes to delimiting the boundaries between taxa. Pheromone trapping, on the other hand, is a way of collecting specimens in a relatively specific fashion while learning important aspects of the communication system of the animal species collected, since pheromone divergence can be an essential element of speciation. There is, as we have shown previously already, a lot to gain by combining the two methods (Svensson et al. 2013).

A major difference to make the distinction between ecological and non-ecological speciation is the order in which reproductive isolation and ecological differentiation take place (Rundell and Price 2009; Svensson 2012). Under non-ecological speciation, reproductive isolation can come first, and ecological differentiation could follow. Under ecological speciation, ecological differentiation comes first and drives reproductive isolation, which is a by-product of niche divergence. In the case of the *Eriocrania* moths, which use birch as exclusive host plant, we propose that reproductive isolation between closely related species could have evolved as the result of divergence in mate preference and the associated pheromone communication system. As these taxa show little differences in their ecology, *Eriocrania* moths may thus represent an example of non-ecological speciation.

## Data Availability

DNA sequences were deposited into Genbank (accession MK456632 - MK457125).
